# Investigating the Role of Polyunsaturated Fatty Acids in Bone Development Using Animal Models

**DOI:** 10.3390/molecules181114203

**Published:** 2013-11-15

**Authors:** Beatrice Y.Y. Lau, Daniel J.A. Cohen, Wendy E. Ward, David W.L. Ma

**Affiliations:** 1 Department of Nutritional Sciences, Faculty of Medicine, University of Toronto, Toronto, ON M5S 1A8, Canada; E-Mail: beatrice.lau@utoronto.ca; 2 Department of Human Health and Nutritional Sciences, College of Biological Sciences, University of Guelph, Guelph, ON N1G 2W1, Canada; E-Mail: cohen@uoguelph.ca; 3 Center for Bone and Muscle Health, Faculty of Applied Health Sciences, Brock University, St. Catharines, ON L2S 3A1, Canada

**Keywords:** polyunsaturated fatty acids, bone mineral density, bone biomechanical strength, nutrition, animal models, growth and development

## Abstract

Incorporating n-3 polyunsaturated fatty acids (PUFA) in the diet may promote the development of a healthy skeleton and thereby reduce the risk of developing osteoporosis in later life. Studies using developing animal models suggest lowering dietary n-6 PUFA and increasing n-3 PUFA intakes, especially long chain n-3 PUFA, may be beneficial for achieving higher bone mineral content, density and stronger bones. To date, the evidence regarding the effects of α-linolenic acid (ALA) remain equivocal, in contrast to evidence from the longer chain products, eicosapentaenoic acid (EPA) and docosahexaenoic acid (DHA). This review reports the results of investigations into n-3 PUFA supplementation on bone fatty acid composition, strength and mineral content in developing animal models as well as the mechanistic relationships of PUFA and bone, and identifies critical areas for future research. Overall, this review supports a probable role for essential (ALA) and long chain (EPA and DHA) n-3 PUFA for bone health. Understanding the role of PUFA in optimizing bone health may lead to dietary strategies that promote bone development and maintenance of a healthy skeleton.

## 1. Introduction

There is an increasing demand to develop food products enriched or fortified with n-3 polyunsaturated fatty acids (PUFA) due to their numerous reported health benefits [1]. Although many of these products are marketed to and consumed by children, the majority of research investigating the effects of n-3 PUFA on growth has focused on visual acuity and neurodevelopment [2,3,4]. In contrast, the potential benefits of these fatty acids on bone outcomes from the early stages of the life cycle through infancy and adulthood is poorly understood. There is emerging evidence for differential effects of specific n-3 PUFA on bone [5], with longer chain PUFA potentially having stronger effects than α-linolenic acid (ALA), which has implications for selecting the appropriate enriched product by the consumer. Furthermore, the complex interplay between n-3 and n-6 PUFA is often simplified into a good *vs*. bad fat relationship, respectively. This simplistic and negative view of n-6 PUFA may not be warranted. Therefore, the purpose of this review is to examine the evidence to date on the effects of n-3 and n-6 PUFA on bone and to critically identify gaps in our understanding.

### 1.1. Bone Development and Peak Bone Mass

Postnatal skeletal development parallels body growth. During growth, lengthening of long bones and strengthening of muscles stimulate bone metabolism [6]. The ultimate goal of altering bone mineral mass and architecture is to enhance bone strength. Skeletal growth accelerates during puberty, and the accumulation of bone mineral reaches its maximum in late adolescence or early adulthood [7]. Commonly referred to as peak bone mass (PBM), this amount of bone mineral is reached by young adulthood and declines in late adulthood. Full genetic potential of PBM is achieved through a combination of lifestyle factors such as physical activity and diet during development [8,9]. There is evidence to show that achieving greater bone mass and strength through diet or exercise in adolescence has benefits on the skeleton that persist into adulthood [10]. Therefore, a higher PBM may delay the increased risk of fracture during aging. In addition, the structure of bone is an important determinant of bone strength [11]. There are some data to suggest that a diet higher in n-3 PUFA and lower in n-6 PUFA modifies fatty acid composition (Table 1) and may favourably promote bone development as measured through changes in bone mineral content, density or bone strength (Table 2 and Table 3).

**Table 1:** Effect of PUFA on bone fatty acid composition in growing animals.

Study subject and age	PUFA source	Amount of PUFA	Control group	Treatment duration	Skeletal site	Outcome on fatty acid composition	Reference
Male quails (1–8 months)	Menhaden oil	n-6/n-3 ratio: 0.66	Soy bean oil n-6/n-3 ratio: 12.6	7 months	Tibia cortical bone	▪ n-6/n-3 ratio: 1.5 in FO, 16.3 in soybean oil ▪ Higher n-3, lower n-6 ▪ Lower ALA, higher EPA and DHA ▪ Lower LA, AA	[12]
Female rats (0–12 weeks)	ALA (flaxseed oil) group (n-3 adq), ALA+DHA (DHASCO oil) group	Total n-3 PUFA: 3.12% in n-3 adq group, 3.83% in n-3 supp group	LA group (n-3 def), high LA group (n-3 def) (LA from safflower oil)	Dams exposed to diet 5 wks before conception. Pups exposed until 12 wks old	Femur cortical bone (results similar in tibia but not shown)	▪ LC n-6/n-3 ratio: 4.7 in ALA+DHA, 6.3 in ALA, 23.4 in LA, 86.6 in High LA ▪ Higher n-3 in ALA and ALA+DHA, lower n-6 in High LA ▪ Higher DHA and ALA in ALA and ALA+DHA; trace EPA; DHA higher than ALA ▪ Lower AA in ALA+DHA	[13]
Male rats (3–9 weeks)	Mixture of safflower oil and menhaden oil	n-6/n-3 ratio: 1.2, 2.6, 9.8, 23.8	N/A	6 weeks	Femur periosteum polar lipids	▪ LC n-6/n-3 ratio: 1.0, 1.4, 2.2, 2.7 ▪ Higher n-3, lower n-6 ▪ Higher EPA and DHA ▪ Lower LA, AA	[14]
					Femur cortical polar lipids	▪ LC n-6/n-3 ratio: 2.0, 2.6, 5.6, 7.1 ▪ Higher n-3, lower n-6 ▪ Higher EPA ▪ Lower AA	[14]
Male and female rats (7–15 weeks)	AIN-93G for n-3 adequate group, Flaxseed oil and DHA for repletion group (n-6/n-3 ratio	n-3 adequate group, n-3 repleted group	Safflower and coconut oil for n-3 depleted group n-6/n-3 ratio: 37	8 weeks	Femur, tibia cortical bone	▪ Femur n-3 in repleted group similar to n-3 in adequate group by 8th week ▪ Tibia more responsive to repletion than femur ▪ Femur and tibia n-6/n-3 in repleted group similar to n-6/n-3 in adequate group by 4th week	[15]
Male and female *Fat-1* mice (0–12 weeks)	Modified AIN-93G, *Fat-1* mice produce n-3 from n-6 in safflower oil	10% w/w safflower oil	Age matched wild-type mice as controls	12 weeks	Femur	▪ Lower n-6/n-3 ratio in *fat-1* mice ▪ Lower n-6, higher n-3 total in *fat-1* mice ▪ DHA highest n-3 in *fat-1*	[16]
					Lumbar Vertebrae 5-6	▪ Lower n-6/n-3 ratio in *fat-1* ▪ *Fat-1* higher total n-3 PUFA than wild-type, higher % of ALA, EPA and DHA	[16]
Male rats (7–16 weeks)	Flaxseed oil for n-3 group, safflower oil for n-6 group	20% w/w flaxseed oil, n-6/n-3: 0.21	Chow diet: n-6/n-3: 9.46	9 weeks	Femur bone marrow, diaphysis, epiphysis (proximal and distal)	▪ Greater level of n-3 PUFA ▪ Predominantly ALA ▪ EPA and DHA not significantly different between treatments ▪ AA not significantly different	[17]
Male rats (0–15 weeks)	AIN-93G with flax for n-3 adequate group, DHASCO for DHA group	ALA 3.1% total lipids, n-6/n-3:5, DHA group 1% total lipids DHA, n-6/n-3: 14.2	Safflower oil (n-6/n-3: 383.7)	15 weeks	Femur and tibia bone marrow and periosteum	▪ ALA diet enriched ALA in marrow and periosteum ▪ ALA feeding resulted in similar DHA composition as DHA fed rats ▪ Similar results in tibia and femur	[18]
Male and female rabbits	Soybean oil (LA/ALA), sesame oil (LA), fish oil (EPA &DHA), algae oil (DHA&AA)	7% w/w oils in diet, soybean oil n-6/n-3: 8.68, fish oil: n-6/n-3: 0.39, algae oil: n-6/n-3: 0.63	Sesame oil n-6/n-3: 21.75	14 weeks	Tibia, femur and humerus bone marrow	▪ Fish oil had lowest n-6/n-3 in bone marrow , followed by algae, soybean and sesame oils ▪ Soybean oil did not produce appreciable amounts of EPA or DHA in bone marrow	[19]

**Table 2:** Effect of ALA on bone mineral and biomechanical strength in developing animals.

Study subject and age	PUFA source	Amount of PUFA	Control group	Treatment duration	Bone mineral	Biomechanical strength	Reference
Female rats (terminated at 30 weeks of age)	Flaxseed oilSoybean oil	n-6/n-3 ratio: 0.4 for n-3 group, 9 for n-6 + n-3 group	Sunflower seed oil (n-6 group) n-6/n-3 ratio: 216	31 days (last 10 days of gestation and first 3 weeks of lactation)	▪ Higher femur mid-diaphyseal BMD in n-3 group and n-6 + n-3 group	▪ Higher cross sectional moment of inertia and moment of resistance at femur mid-diaphysis in n-6 + n-3 group	[20]
Male and female mice (4–13 weeks)	Flaxseed oil	10% flaxseed oil n-6/n-3 ratio: 0.25	10% corn oil n-6/n-3 ratio: 57	9 weeks	▪ No difference in femur and lumbar vertebrae BMC and BMD	▪ No difference in peak load, yield load, resilience, toughness, and stiffness at femur midpoint ▪ No difference in peak load at femur neck and lumbar vertebrae	[21]
Male piglets (5–26 days)	Soybean, safflower, coconut, and flaxseed oils	n-6/n-3 ratio: 4.5	n-6/n-3 ratio: 9.0	21 days	▪ No difference in femur, lumbar vertebrae, and whole body BMC	▪ Not tested	[22]
Female rats (4–12 weeks)	Flaxseed oil	Flaxseed oil (12% w/w) n-6/n-3 ratio: 0.33	Corn oil n-6/n-3: 73	8 weeks	▪ No difference in tibia or femur BMD or BMC	▪ No differences in peak load, stiffness, bending stress in tibia or femur	[23]
Female chickens (16 weeks to 58 weeks)	Flaxseed oil	Varying amounts of flaxseed oil and corn oil in diet n-6/n-3: 47.8–4.8	N/A	42 weeks	▪ No difference in tibia BMD or BMC	▪ No effect on ultimate stress, bending strain, or Young’s modulus of elasticity in tibia	[24]
Male rats (7–16 weeks)	Flaxseed oil	20% w/w flaxseed oil n-6/n-3 ratio: 0.21	Chow diet, n-6/n-3: 9.46	9 weeks	▪ Higher femoral BMD, BMC than chow fed rats	▪ Stiffness and peak load at femur midpoint higher in n-3 group	[17]
Male rats (4–12 weeks)	Flaxseed oil	20% w/w n-6/n-3: 0.4	20% w/w Corn oil n-6/n-3 ratio: 9.0	8 weeks	▪ No difference in total skeleton or spine BMD or BMC	▪ Not tested	[25]
Male rats (conception-7 weeks or conception-19 weeks)	Flaxseed oil	10% ground flaxseed, approx. 4% w/w flaxseed oil	AIN-93G with safflower oil, n-6/n-3: 6	Gestation and lactation, or continuation until 7 weeks or 19 weeks	▪ No difference in BMD or BMC in any of the treatment groups at both time points	▪ Ultimate bending stress and Young’s Modulus lower at 7 weeks in flaxseed fed group ▪ No differences in any measure of biomechanical strength at 19 weeks	[26]

**Table 3:** Effect of long chain PUFA on bone mineral and biomechanical strength in developing animals.

Study subject and age	PUFA source	Amount of PUFA	Control group	Treatment duration	Bone mineral	Biomechanical strength	Reference
Male quails (1–8 months)	Menhaden oil	n-6/n-3 ratio: 0.66	Soy bean oil n-6/n-3 ratio: 12.6	7 months	▪ Higher tibia BMC	▪ Higher sheer force and shear stress at the tibia ▪ Higher cortical density at distal and proximal ends of tibia ▪ Higher cortical thickness at diaphysis and proximal ends of tibia	[12]
Male rats (4–9 weeks)	Menhaden oil	4% menhaden, 3% corn—7% w/w diet n-6/n-3 ratio: 1.4	AIN-93G with 7% soybean oil n-6/n-3 ratio: 7.1	5 weeks	▪ Higher femur BMD	▪ Not tested	[27]
Male mice (6–12 weeks)	Tuna oil	4% tuna oil + 1% corn oil n-6/n-3 ratio: 0.5 26.3% DHA	5% corn oil n-6/n-3 ratio: 45.3	6 weeks	▪ Higher lumbar spine BMD ▪ Higher femur BMD	▪ No difference at femur midpoint	[28]
	Fish oil	4% fish oil + 1% corn oil n-6/n-3 ratio: 0.5 11.7% DHA			▪ No difference in femur and lumbar spine BMD	▪ No difference at femur midpoint	[28]
Male and female rats (3–8 weeks)	Menhaden oil	6% menhaden oil + 1% soybean oil	7% soybean oil	5 weeks	▪ No difference in femur and lumbar vertebrae BMC or BMD	▪ No difference in strength at femur midpoint ▪ Lower peak load at lumbar vertebra in female rats ▪ No difference in peak load of lumbar vertebra in males	[29]
Male and female rats (7–15 weeks)	AIN-93G for n-3 adequate group, DHASCO for repletion group	Repletion diet: AIN-93G with DHASCO (3.0 g/kg diet) ALA: 2.6% of total fatty acids DHA: 1.3% of total fatty acids n-6/n-3 ratio: 4	Safflower and coconut oil for n-3 depleted group n-6/n-3 ratio: 378	8 weeks	▪ Not tested	▪ At wk 0, tibia of adequate group had higher load at failure, peak load, bending moment ▪ At wk 8, tibia of repleted group had higher ultimate stress ▪ At wk 8, tibia of adequate group had higher energy to peak load ▪ No differences in biomechanics of femur	[15]
Male rats (0–15 weeks)	DHA (DHASCO, ALA from Flaxseed oil	Flaxseed ALA 3.1% total lipids, n-6/n-3:5, DHA group 1% total lipids DHA, n-6/n-3: 14.2	Safflower oil (n-6/n-3: 383.7)	15 weeks	▪ Femur BMC correlated with total n-3 PUFA ▪ No association seen with tibia	▪ Not tested	[18]
Female rats (4–12 weeks)	Krill oil	12% w/w diet, n-6/n-3: 0.03	Corn oil, 12% w/w diet	8 weeks	▪ No difference in femur and tibia BMC or BMD	▪ No difference in femur or tibia biomechanical strength	[23]
	Salmon Oil	12%w/w diet, n-6/n-3: 0.04			▪ Higher tibia BMC than control	▪ No difference in femur or tibia biomechanical strength	[23]
	Tuna Oil	12%w/w diet, n-6/n-3: 0.04			▪ Higher tibia BMC and BMD than control	▪ No difference in femur or tibia biomechanical strength	[23]
	Menhaden oil	12%w/w diet, n-6/n-3: 0.04			▪ No difference in BMD and BMC	▪ No difference in femur or tibia biomechanical strength	[23]
Female rats (0–21 weeks)	Menhaden oil	6.5% w/w menhaden oil diet, 1% safflower oil, n-6/n-3: 0.5	Safflower oil 7.5% w/w diet, n-6/n-3: 250	21 weeks	▪ Not tested	▪ Menhaden oil: higher maximum force to break femur by three point bending ▪ Menhaden oil: higher force for compression of vertebra	[30]
Male rats (8–10 weeks)	Fish oil	15% w/w diet as fish oil, n-6/n-3: 0.52	15% w/w diet soybean oil, n-6/n-3: 11.7	15 days	▪ Not tested	▪ Fish oil: higher maximum force to break tibia by three point bending ▪ Fish oil: higher stiffness, resilience and absorbed energy	[31]

### 1.2. Dietary Polyunsaturated Fatty Acids (PUFA)

**Figure 1:**
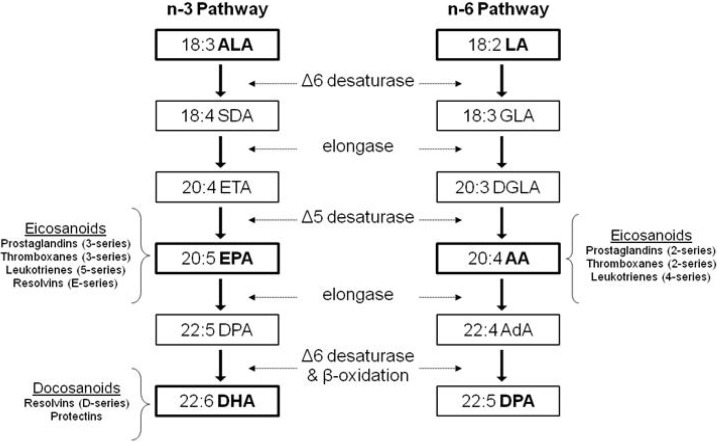
Synthetic Pathways of PUFA. α-linolenic acid (ALA; 18:3n-3) and linoleic acid (LA; 18:2n-6) are essential PUFA obtained from the diet, and are substrates for elongase and desaturases for the synthesis of long chain, more unsaturated PUFA eicosapentaenoic acid (EPA; 20:5n-3), docosahexaenoic acid (DHA; 22:6n-3), and arachidonic acid (AA; 20:4n-6). Relevant intermediates in these pathways include SDA (stearidonic acid), ETA (eicosatetraenoic acid), DPA (docosapentaenoic acid), GLA (γ-linolenic acid), DGLA (dihomo-γ-linolenic acid) and AdA (adrenic acid). These pathways employ the same enzymes, however they are not interconvertible in mammals, *i.e.*, n-6 PUFA cannot be converted into n-3 PUFA and vice versa. AA and EPA are substrates for eicosanoid production, and both compete for the same enzymes. DHA can be metabolized to docosanoids. The products of n-6 PUFA tend to exert inflammatory effects while the products of n-3 PUFA tend to be anti-inflammatory.

PUFA are fatty acids that contain more than one *cis*-double bond that are separated by a methylene group. n-3 and n-6 PUFA are the two major families of PUFA which differ in the location of the first double bond counting from the methyl terminus. Mammals are unable to synthesize n-3 PUFA and n-6 PUFA due to the lack of appropriate desaturases. Therefore, α-linolenic acid (ALA; 18:3n-3) and linoleic acid (LA; 18:2n-6) are essential PUFA that must be obtained in the diet. ALA and LA are found abundantly in plant-based oils. Major food sources of ALA are flaxseed, canola and soybean oils, while safflower, sunflower and corn oil are major food sources of LA. ALA is typically found in low concentrations in most mammalian cell membranes, suggesting that its direct effects on cell and tissue function may be limited [32]. However, both ALA and LA are substrates for elongase and desaturases for the synthesis of longer chain PUFA, eicosapentaenoic acid (EPA; 20:5n-3), docosahexaenoic acid (DHA; 22:6n-3), and arachidonic acid (AA; 20:4n-6) (Figure 1) [12]. The rate of conversion from ALA to EPA is estimated at 0.2%–8%, and full conversion to DHA at 0.05%–4%, with diet and gender playing a significant role [32]. EPA and DHA are also naturally present in seafood and fish oils. It is important to note that both pathways employ the same enzymes. Thus, the relative amount of n-3 and n-6 PUFA consumed in the diet can affect the conversion rate of either fatty acid through competitive binding of the shared metabolic enzymes.

Cell membranes are composed of phospholipids consisting of phosphatidylcholine (PC), phosphatidylethanolamine (PE), phosphatidylinositol (PI), and phosphatidylserine (PS). PC and PE are major components of membrane bilayers. PI and PS in particular are known to play important roles in cell signalling and eicosanoid synthesis [33]. PUFA incorporated into membrane phospholipids can be cleaved by phospholipase A_2_ (PLA_2_), releasing AA, EPA, and DHA. AA and EPA are substrates for eicosanoid production, and both compete for the same enzymes. AA leads to the production of eicosanoids including prostaglandin-2- (*i.e.*, PGE_2_), thromboxane-2-, and leukotriene-4-series, while EPA gives rise to eicosanoids of prostaglandin-3-, thromboxane-3-, leukotriene-5-series, and E-series-resolvins [34]. DHA can be metabolized to docosanoids such as D-series-resolvins and protectin. Production of eicosanoids from n-6 PUFA usually exert inflammatory effects while the products of n-3 PUFA exert anti-inflammatory effects [35]. Mice fed diets rich in n-6 PUFA led to the production of higher amounts of AA-derived eicosanoids compared with diets with a lower n-6/n-3 ratio [36]. While normal levels of PGE_2_ are required for cellular and physiological functions, *in vitro* evidence shows high levels of PGE_2_ exert inflammatory effects such as stimulating the production of inflammatory cytokines such as IL-6 and pro-inflammatory 4-series leukotrienes [35]. PGE_2_ also has anti-inflammatory effects demonstrated *in vivo*, such as the formation of anti-inflammatory lipoxins (LXA_4_) [37]. AA-derived leukotriene B_4_ (LTB_4_) upregulates the synthesis of pro-inflammatory cytokines such as TNF-α, IL-1, and IL-6, whereas LTB_5_ derived from EPA can inhibit the synthesis of the pro-inflammatory LTB_4_. Inflammation is also reduced by E-series resolvins as they suppress the activation of nuclear factor kappaB (NFκB), the central transcription factor involved in the mediation of inflammatory responses, and the consequent synthesis of proinflammatory cytokines, as shown in human endothelial cells [38]. In addition to effects on the immune system and inflammation, PUFA have also been shown to become enriched in bone tissue (Table 1) and affect cellular processes in bone metabolism that are discussed further below.

## 2. n-3 PUFA and Its Effect on Bone Fatty Acid Composition

To determine a role for n-3 PUFA in bone metabolism, an important first step is to understand how dietary PUFA modifies bone fatty acid composition. In particular, whether there are different effects attributed to specific n-3 PUFA, *i.e.*, ALA *vs.* longer chain EPA and DHA. Bone fatty acid composition has been studied after conventional dietary supplementation and using the novel transgenic *fat-1* mouse to understand the responsiveness of bone tissue to changes in dietary fatty acids or their metabolism.

### 2.1. ALA Studies

Although much attention is given to the beneficial health effects of longer chain EPA and DHA on bone metabolism, health effects of ALA are much less understood. This is important because the majority of dietary n-3 PUFA intake in North America is from ALA [5]. Therefore, it is relevant to determine if ALA consumption can have similar effects as EPA and DHA on bone fatty acid composition. Only a few studies have investigated how feeding a diet rich in ALA alters bone health in developing animals [13,17,18,26]. Li *et al.* [13] examined the effects of ALA (0.48% w/w flaxseed oil) and LA (8.5% w/w safflower oil) rich diets on bone fatty acid composition in growing female rats for 12 weeks, with both diets receiving 10% w/w total fat. Compared to the high LA group, the ALA group had a significantly lower ratio of n-6/n-3 in the femoral cortical bone, specifically enriched in ALA and DHA. Although DHA was enriched with ALA feeding, EPA was only present in trace amounts, suggesting that bone specifically incorporates DHA preferentially over EPA. However, the ALA enriched diet did not have an effect on lowering the AA composition in the bone. Similarly, it was demonstrated that newborn rat pups raised on an n-3 adequate diet with ALA, or an n-3 deficient diet with LA as the main PUFA source for 15 weeks resulted in significantly higher levels of ALA and DHA in the femoral periosteum and bone marrow in the adequate group [18]. It was demonstrated that high fat diets rich in ALA and LA as 20% w/w of diet, resulted in modification of tissue fatty acid composition mirroring the diets in both bone marrow and femur diaphysis [17]. However, there were no discernible changes in the composition of EPA, DHA or AA, suggesting that increasing fat content in the diet may have had a negative impact on the conversion of ALA and LA to their longer chain products. Overall, these studies collectively show that feeding ALA results in selective enrichment of DHA compared to EPA in bone.

### 2.2. EPA and DHA Studies

Feeding fish oil or other sources of long chain PUFA in the diet also promotes changes in the fatty acid environment within bone compartments that is reflective of the diet [12,13,14,19]. Feeding growing rats diets containing menhaden oil as 10%–70% of total fat dose-dependently increased both EPA and DHA within femur bone marrow and periosteum [14]. The selective incorporation of DHA rather than EPA into bone is also evident in fish oil fed animals. Rabbits fed fish oil (EPA and DHA, 1.4% w/w 2.1% w/w respectively) showed significantly greater incorporation of DHA than EPA into bone, relative to the diet [19].

EPA and DHA can also be produced through genetic manipulation. The *fat-1* mouse model has become an important tool in n-3 PUFA research. The *fat-1* mouse is a transgenic model expressing a humanized form of the *C. elegans fat-1* gene, which encodes an n-3 fatty acid desaturase. This allows transgenic animals to endogenously synthesize n-3 from n-6 PUFA, eliminating the need to obtain n-3 PUFA from the diet [39]. To our knowledge, only one developmental study has examined the lipid profile of lumbar vertebrae after exposure to n-3 PUFA, which was in the *fat-1* mouse model [40]. Femoral and vertebral phospholipids showed higher n-3 to n-6 ratio in transgenic mice [16]. The lumbar spine has a higher proportion of trabecular bone, which has a higher turnover rate and is more metabolically active, than cortical bone. We revealed that in wild-type and transgenic mice that were able to convert n-6 to n-3 PUFA, the n-6/n-3 ratio in the lumbar vertebrae seemed to be lower than that in the femur [16]. Furthermore, the PUFA compositions in lumbar vertebrae had a greater degree of correlation with BMD and biomechanical strength compared to femur, although femur BMD was significantly correlated with EPA and DHA. Since this study employed a genetic approach rather than conventional dietary manipulation, it would be worthwhile to determine if this result can be duplicated in feeding studies, since the *fat-1* mouse compares very well to fish oil fed mice, having similar amounts of EPA and DHA in tissues [41].

### 2.3. Effect of ALA *vs.* EPA and DHA

A number of studies have compared the effects of feeding ALA to EPA and/or DHA to discern if there are differences in fatty acid content in bone [12,13,14,15] (Table 1). Feeding fish oil to growing male quails resulted in higher levels of EPA and DHA in tibial cortical bone relative to soybean oil (containing 7% of fatty acids as ALA) fed quails [12]. In a rat study, ALA as 0.3% of the diet was needed to achieve the same level of DHA in bone as rats fed DHA as 0.1% of the diet [18]. In another study [13], female rats were exposed to ALA+DHA, ALA, LA, or high LA from five weeks before conception, as the corresponding diets were also fed to the dams, to 12 weeks of age. ALA and ALA+DHA groups had higher ALA and DHA in bone and lower total n-6 PUFA than LA and high LA groups, although AA levels were only lower in the ALA+DHA group. DHA levels were higher than ALA levels in all the groups, again suggesting that DHA is preferred in bone tissue [13]. The level of DHA achieved by feeding rats ALA alone was not as high as feeding rats both ALA with DHA. Overall, feeding ALA resulted in less DHA incorporation into bone as compared to feeding preformed DHA from fish oil. It is evident from these studies that direct feeding of fish oil or DHASCO containing the preformed fatty acids is more efficient in the incorporation of EPA and DHA into bone. Additionally, in the studies examined, fish oil tends to have the effect of lowering AA content in bone, whereas this effect is blunted or absent in flaxseed or soybean fed animals. These differences in incorporation into tissue with different n-3 sources may explain the discrepancies in the functional outcomes discussed in the next section.

## 3. PUFA and Bone Mineral Content and Strength

### 3.1. ALA Studies

In addition to studies demonstrating that changes in dietary fatty acid composition are reflected in bone tissue, studies have examined the effects of ALA alone on bone development, which have yielded mixed results [17,20,21,23,24,25,42] (Table 2). Thirty-week-old female rats exposed to flaxseed oil or soybean oil from the last ten days of gestation to three weeks postnatal had greater BMD at the femur mid-diaphysis than their littermates fed sunflower seed oil [20]. Rats fed the soybean diet also had higher cross sectional moment of inertia and moment of resistance, indicating that the femur was more resistant to bending and torsion, respectively. However, in young male and female mice fed a flaxseed oil diet from 4 to 13 weeks of age for 9 weeks, no differences were found in their BMD and biomechanical strength properties at the femur or the lumbar vertebrae compared to mice fed a corn oil diet [21]. Male rats fed a 4% w/w flaxseed oil diet had a lower measure of Young’s modulus and bending stress (measures of bone strength) after 7 weeks compared to those on the basal diet without flax, however these differences did not persist through adulthood [26]. Various levels of soybean, safflower, coconut, and flaxseed oil fed to male piglets from postnatal day 5 through 26 had no effect on femur lumbar vertebrae, and whole body BMC [42]. The n-6/n-3 ratio of the diets in this study was between 4.5 and 9.0. This is in agreement with a study on growing chickens fed a range of diets containing a n-6/n-3 ratio of 47.8-4.8, which did not find a significant difference in BMC or any measure of biomechanical strength [24]. A few studies have also examined the additional influence of high fat diets containing ALA on bone properties in developing animals. A study performed on growing male rats [17], 6–15 weeks of age, compared a high fat diet (20% wt/wt lipids from ALA-rich flaxseed oil) to a standard chow diet (16% wt/wt lipids). The flaxseed group had greater BMD and BMC, as well as improved markers of biomechanical strength such as higher peak load at the femur midpoint. However, another study that utilized a 20% fat diet from flaxseed oil, rich in ALA, did not show any effect on BMD, BMC or biomechanical strength in growing male rats from 3–11 weeks of age, relative to corn oil fed rats [25]. Overall, due to the limited number of studies showing a weak or absent effect of ALA, it is not possible yet to conclude a definitive role for ALA in bone health. However, despite these conflicting results, these studies demonstrate no adverse effects of ALA.

### 3.2. EPA and DHA Studies

In contrast to flaxseed and soybean fed animals, studies involving marine sources containing EPA and DHA have more consistently shown a positive role on BMC, BMD and bone strength. As summarized in Table 3, feeding fish oil results in greater BMC, BMD and/or biomechanical bone strength in various growing animal models. Growing quails fed menhaden oil demonstrated higher tibial BMC, as well as higher measures of tibial strength compared to those fed soybean oil [12]. Additionally, mice fed menhaden oil as 4% w/w diet had higher femoral BMD than those fed soybean oil [27]. These studies show that feeding EPA and DHA improved both measures of strength and BMD.

One study investigated different marine sources of EPA and DHA. Male mice fed tuna oil (4% w/w), had greater femur and lumbar vertebra BMD than mice fed a corn oil diet rich in n-6 PUFA [28]. However, no effect on BMD at either site was observed for a different fish oil treatment in this experiment, which contained less DHA than the tuna oil diet.

Comparing the effects of different marine oil sources with similar EPA and DHA content suggests potential oil specific effects [23]. Only tuna and salmon oil, but neither menhaden nor krill oil improved BMD and BMC in tibia, suggesting a role for DHA specific oils. Feeding a DHA rich diet containing DHASCO (an oil fortified with DHA from an algal source, approximately 40% DHA, 40%–60% saturates, 10%–20% monounsaturates) for eight weeks to rats that were previously consuming a diet deficient in n-3 PUFA had greater biomechanical strength in the tibia than rats maintained on the n-3 deficient diet [15]. While more research is needed, these studies suggest a DHA specific effect on bone BMC, BMD and strength.

One study [29] reported after feeding weanling rats 6% (w/w) menhaden oil for 5 weeks, females showed a lower peak load at the 5th lumbar vertebra, as well as lower femur length. There were no differences in any measure of bone strength or BMD in the males in this study. These results suggest that although the consensus appears to be positive in terms of incorporating n-3 PUFA into diet, further research is needed to confirm there are no adverse effects of consuming high amounts of EPA and DHA early in life.

### 3.3. Comparison of ALA *vs.* EPA/DHA Studies

Comparing the results of studies involving ALA with studies using EPA and DHA, ALA appears to have a lesser effect than their long chain products. Although it is possible that ALA indirectly exerts its effects through its conversion into EPA and/or DHA, the likelihood or the effectiveness of this mechanism relies on the rate of conversion, which is known to be low in humans [32]. However, the conversion of PUFA is also known to be species and tissue specific, with rodents possessing a higher capacity to convert ALA and LA to their long chain products [43]. In the studies using ALA described in Table 2, there are differences in the methodology that may explain the conflicting findings. Feeding rats an ALA rich diet in utero demonstrated positive results [20], whereas feeding similar ALA-rich diets post-natally did not confer any positive benefits [21,42]. Therefore, it is possible that any action of ALA on bone health may be programmed as early as during gestation, and ALA may exert its potentially favourable effects during a limited developmental window. While further research is needed, the available data indicates a potentially beneficial effect of n-3 PUFA, especially DHA. Moreover, the potential benefit of ALA rich diets *vs.* traditionally termed “unhealthy” fats should be studied.

## 4. Mechanisms of PUFA in Bone Metabolism

**Figure 2:**
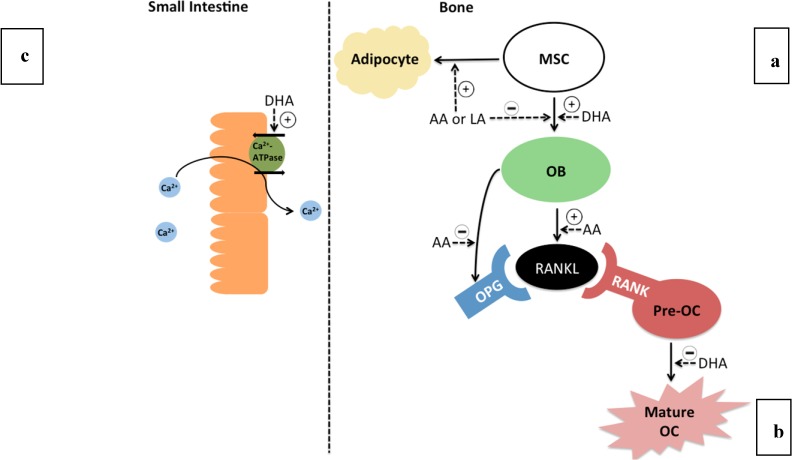
Potential mechanisms linking PUFA intake and bone metabolism. (**a**) n-3 and n-6 PUFA modulate differentiation from mesenchymal stem cells (MSC) in bone. n-6 PUFA (arachidonic acid; AA and linoleic acid; LA) stimulate adipogenesis, as well as inhibit osteoblastogenesis at high levels. n-3 PUFA (docosahexaenoic acid; DHA) has been shown to upregulate osteoblast (OB) maturation in comparison to n-6 PUFA. The increased osteoblast population may lead to stronger bones. (**b**) AA leads to the increased production of soluble and membrane bound receptor-activated nuclear kappa B ligand (RANKL) by osteoblasts, which binds to its receptor RANK on pre-osteoclasts causing maturation into activated osteoclasts (OC). Therefore, higher AA stimulates osteoclast differentiation. Osteoprotegrin (OPG) is a decoy receptor for RANKL produced by osteoblasts, which can be lowered by AA, therefore increasing RANK activation. DHA has been shown to inhibit osteoclast maturation, and EPA/DHA may indirectly prevent AA stimulated RANKL production by lowering membrane derived AA. (**c**) n-3 PUFA may modulate absorption of Ca^2+^ by activating transporters in the small intestine.

Although no all-encompassing mechanism exists to connect PUFA and bone health, several potential theories exist to explain the beneficial effects of n-3 PUFA on bone metabolism (Figure 2). One such mechanism is that n-3 PUFA can increase calcium absorption in the small intestine. In growing rats fed either sunflower oil or fish oil for 12 weeks, fish oil fed rats displayed increased calcium balance and increased absorption by the small intestine [44]. Enterocytes isolated from rat small intestine were exposed to EPA, DHA or AA, and it was demonstrated that only DHA was able to increase basal calcium absorption by increasing the activity of Ca-ATPase, which represents the rate limiting enzyme in calcium uptake [45]. By increasing calcium absorption, more calcium is available to be incorporated into the bone mineral matrix. Higher calcium absorption was similarly shown in rats fed tuna oil and was correlated to higher BMD [23].

n-3 PUFA also have the potential ability to affect bone marrow cells early in development. It was shown that DHASCO feeding of young weanling rats (3 weeks) increased bone marrow cellularity [46]. Bone marrow contains mesenchymal stem cells (MSCs) that have the potential to differentiate into adipocytes or osteoblasts [47]. Therefore, depending on the developmental fate of MSC, these cells can either contribute to bone marrow adiposity by undergoing adipogenesis, or increase bone mass or BMD by increasing osteoblastogenesis. Both n-6 and n-3 PUFA are ligands for PPARγ2, a transcription factor that activates adipogenic genes. In cultured human MSC stimulated to differentiate into adipocytes or osteoblasts, DHA treatment had no effect on adipogenesis, but a small effect on stimulating osteoblast differentiation [47]. Addition of EPA and DHA increased markers of mineralization, and thus stimulated the differentiation of MSCs to osteoblasts. In contrast, AA was a potent stimulator of adipogenesis and PPARγ activity. AA additionally inhibited differentiation to osteoblast compared with DHA treated cells. Thus, decreasing the n-6/n-3 ratio in bone may indirectly have the effect of increasing osteoblast activity, leading to increased bone formation early in life.

While the products of n-6 PUFA, such as AA, are generally regarded as pro-inflammatory, these fatty acids also play a role in normal bone development [14]. AA, the main n-6 PUFA cleaved from membrane phospholipids, becomes a substrate of COX for the production of PGE_2_. At physiological levels, PGE_2_ stimulates normal bone growth [14,48]. However, at higher levels, PGE_2_ stimulates osteoblasts to express the protein receptor activator for nuclear factor ß ligand (RANKL). RANKL binds to its receptor (RANK) on osteoclast precursors, and stimulates differentiation into mature and activated osteoclasts. Greater level of incorporation of n-3 PUFA into cell membranes displaces AA from membrane phospholipids. Thus, increasing n-3 PUFA in the diet, particularly long chain n-3 PUFA, may reduce production of AA derived PGE_2_ and subsequent osteoclastogenesis. In tandem, EPA is a substrate for PGE_3_, and while PGE_3_ also promotes osteoclastogenesis, the conversion of EPA to PGE_3_ is much less efficient, resulting in lower levels of PGE_3_ [49]. In addition, osteoblasts produce osteoprotegerin (OPG), a decoy receptor for RANKL. OPG binds membrane-bound or soluble RANKL produced by osteoblasts and results in the inhibition of the RANK-RANKL interaction and maturation of osteoclasts. Exposing cultured osteoblast-like cells to AA inhibited production of OPG [50].

In weanling 3 week old rats, consumption of fish oil intake for an additional 6 weeks resulted in lower *ex vivo* PGE_2_ production in bone [14]. However, one study did not observe any differences in PGE_2_ released from the tibia of 26-day-old male piglets that were fed different n-6/n-3 ratios using flaxseed oil diet as a source of ALA for 21 days. This suggests differential effects between ALA and EPA/DHA on PGE_2_ [27]. Male rats that received DHA from dams fed a 1% DHA (w/w, from DHASCO) diet had higher osteoblast density and lower osteoclast activity at three weeks of age than rats fed 0.1% DHA, as measured histologically [51]. These markers coincided with greater bone mass at three weeks. However, after weaning at 3 weeks to a chow diet, these benefits did not persist to 6 or 12 weeks.

This specific mechanism has been explored using cell culture. Exposing cultured osteoblast-like cells to DHA treatment resulted in RANKL expression below detection limits, while AA treatment increased expression of RANKL [50]. Furthermore, treatment of RAW 264.7 cultured cells with DHA caused a reduction in osteoclastogenesis as compared to cells treated with LA [52]. Therefore, n-3 and n-6 PUFA may have opposing effects on osteoclastogenesis. Collectively, these studies show a role in n-3 PUFA in reducing osteoclast activity.

The type of n-3 PUFA should be considered as they may have different effects on bone formation and resorption. In developing rats, feeding a menhaden oil diet resulted in higher alkaline phosphatase (ALP), a bone formation marker, and a higher rate of bone formation was confirmed histologically. In contrast, studies that used ALA as the source of n-3 PUFA resulted in more subtle effects on rates of bone formation or bone resorption [22,42]. Additional studies are needed to more fully understand the extent to which individual n-3 PUFA and n-6 PUFA modulate bone development, and influence BMD, BMC and bone strength. In tandem, elucidating mechanisms of action is also warranted.

## 5. Knowledge Gaps and Future Directions

The evidence presented in this review provides a probable role for EPA and DHA in bone health during development, while the function of ALA remains poorly understood. Although generally regarded as pro and anti-inflammatory, n-6 and n-3 PUFA, respectively, appear to have effects in bone metabolism that exist outside these stereotypical roles. In general, there remains much to be understood about the individual effects of specific n-6 and n-3 PUFA. To further refine our understanding of fatty acids and bone development, there are a number of knowledge gaps needing further study which are summarized in Table 4.

**Table 4:** Summary of Knowledge Gaps and Future Directions.

Research Area	Current State	Knowledge Gap	Future Directions
Dose and Type of PUFA	Providing n-3 PUFA to deficient animals corrects deficiency Varying amounts of fatty acids in diet have had beneficial effects on bone health, but results in inconsistent findings based on levels of fatty acid in the diet (Table 2 and Table 3)	What is the normal range of fatty acids (ALA, EPA, DHA, LA, AA) in the diet needed for optimal bone development? Is there any extra benefit/detriment to increasing n-3 past adequacy? What is an appropriate control diet? Animal studies typically involve only n-3 and n-6 PUFA fed groups. Thus, how do the effects of n-3 and n-6 compare to saturated and monounsaturated fats? Are n-6 PUFA really bad or only relative to n-3 PUFA? Does the amount of fat in the diet influence bone outcomes?	Dose response studies in similar animals (low, medium, high, super-physiological) Compare effects of individual n-3 (ALA, EPA & DHA) and n-6 PUFA (LA and AA) Compare effects on a background diet consisting of low or high fat Incorporate the use of saturated and monounsaturated fat diets as control diets relative to n-3 and n-6 PUFA diets
Timing of Exposure	Comparing studies of in utero and perinatal (during late pregnancy and lactation) exposure seems to show greater effect of PUFA, specifically ALA, on bone than post-natal exposure n-3 PUFA exposure in utero and during lactation confer bone benefits to young animals, but may not persist once supplementation ceases	What is the optimal time period to consume n-3 PUFA? What is the optimal duration of intake? What mechanisms mediate effects in utero?	Compare intake at different life stages (in utero, post-natal) Compare acute *vs.* chronic exposure of fatty acids Investigate the role of n-3 in developing bone tissue and osteoblastic/adipogenic gene expression at different life stages
Gender Differences	Sex differences in fatty acid metabolism, possible due to sex steroids, but has not been consistently observed	Does dietary PUFA affect bone development in males and females differently? Are there differences in the optimal dose for males and females?	More studies need to include both genders to identify gender specific effects Dose-responses studies are needed in males and females

### 5.1. Dose and Type of PUFA

There are several important methodological and experimental design considerations to be considered in future studies. Chow diets should be avoided as the composition of these diets is generally uncertain. The source of oil (*i.e.*, menhaden *vs.* tuna) and the relative composition of individual n-6 and n-3 PUFA should be carefully considered.

There remains insufficient data to make recommendations regarding n-3 PUFA intake for optimal bone development. Although long chain n-3 PUFA (EPA and DHA) appear to exert a more favourable influence on BMD and biomechanical strength than ALA, the potential bioactivity of ALA requires further investigation. It remains important to examine individual PUFA and their combined effects on bone growth in healthy animals. Many food products in the market are advertised to be fortified with n-3 PUFA without specifying the specific type. If ALA and its long chain products can indeed exert different biological functions on the skeleton, consumers need to be aware of how these products may have an impact on their bone health. The recently developed ∆^6^-desaturase knockout model cannot convert ALA to longer chain EPA and DHA, allowing researchers to delineate fatty acid specific effects [53]. This will be a useful tool in determining what, if any, are the specific biological activities of ALA on bone strength and metabolism. As well, although no convincing outward effects have been demonstrated thus far using ALA rich food sources, perhaps more sensitive techniques for determining bone outcomes are needed to determine the role of ALA in bone health.

### 5.2. Timing of Exposure

As the greatest positive effect seen was observed when ALA treatment was delivered in utero, further research should examine how this benefit is conferred during this developmental window. Of greater interest is whether beneficial bone adaptations such as increased bone mass and strength from PUFA will persist later in life and help reduce fracture risk. However, species-specific differences should be taken into account when using animal models. While mice also attain peak whole body BMD at adolescence (approximately 12 weeks), and similar to humans, lose bone density with aging, not all factors remain the same. In contrast to humans, in both the rat and the mouse, cortical bone continues to grow as the animal grows, and cross-sectional inertia of bone increases as well, which may confound results of aging rodent models [54]. Additionally, there is no fusion of the growth plates in rats and therefore they do not achieve peak bone mass at the same time as mice or humans. For this reason, studies aiming to affect peak bone mass and strength in rodent models should be limited to younger animals (3–4 months), and mice may represent a more appropriate growth model for bone development research [54,55].

It was recently shown in humans that maternal long-chain PUFA status at 34 weeks gestation was a determinant of bone health in offspring. Plasma EPA and DPA during late pregnancy correlated positively with whole body BMD and lumbar BMD of offspring at 4 years of age [56]. This suggests that maternal PUFA status plays a role in bone accrual in childhood, however longer term studies need to be undertaken to confirm this role. Only one cohort study has investigated and reported an association between fatty acid composition of serum phospholipids and BMD in a group of healthy young men from 16 to 22 years of age [57]. DHA was positively correlated with BMD of the whole body and the spine at 22 years of age, and with the change in BMD at the spine between 16 and 22 years of age. However, a recent clinical trial provided an n-3 supplement as 900mg of DHA and200 mg EPA daily to boys aged 13–15 for 16 weeks, and there were no associations between EPA or DHA status and BMC, BMD or markers of bone formation [58]. While the results gathered from animal models thus far would suggest n-3 PUFA intake to be beneficial to bone growth, further large scale clinical trials are needed to ascertain this claim. If the biological functions of n-3 PUFA can be further elucidated, the intake of these oils in utero and/or during infancy, childhood and adolescence may provide an effective prevention against the loss of bone mineral and overall deterioration of bone tissue during aging.

### 5.3. Gender Differences

Some, but not all animal studies have examined PUFA intake and bone development in both genders. Although fracture risk is higher in women due to the factors associated with post-menopause, the lifetime fracture risk for 50-year old males is 20%—a significant threat [59]. Therefore, it is equally important to study both males and females in bone and fracture risk research. There remains more research to be done on the effect of PUFA in both males and females. Evidence suggests that sex hormones play a role in fatty acid metabolism by influencing the enzymatic activity of the PUFA conversion pathways. Estrogen has been shown to have a stimulatory effect on the conversion of essential fatty acids, where testosterone is inhibitory [60] (Figure 1). In human studies, females have been reported as having higher conversion rates of ALA to longer chain fatty acids in isotope tracer studies. However, in studies involving the *fat-1* mouse, male mice showed greater incorporation of n-3 PUFA into femurs than females, suggesting there are gender differences in PUFA metabolism that exist in bone [16]. Therefore, further research is needed to determine these specific mechanisms. Thus, potential recommendations for PUFA intake in regards to bone health may need to be gender-specific.

## 6. Summary and Conclusions

- There is a probable role for n-3 PUFA in the development of stronger bones, with EPA and DHA intervention being more efficacious than ALA. Whether this translates to reduced fracture risk in humans remains to be determined.
- Both n-3 and n-6 PUFA play a role in bone development. Future research should be aimed at determining the dose, duration, and timing of exposure to individual n-3 and n-6 PUFA throughout the lifecycle.
- n-3 PUFA may improve bone health by increasing calcium absorption in the gut, and increasing osteoblast differentiation and activity, reducing osteoclast activity and promoting deposition of mineral in developing bones. These mechanisms require further study.
